# Menopausal Hormone Therapy and Lung Cancer-Specific Mortality Following Diagnosis: The California Teachers Study

**DOI:** 10.1371/journal.pone.0103735

**Published:** 2014-07-31

**Authors:** Jessica Clague, Peggy Reynolds, Katherine D. Henderson, Jane Sullivan-Halley, Huiyan Ma, James V. Lacey, Shine Chang, George L. Delclos, Xianglin L. Du, Michele R. Forman, Leslie Bernstein

**Affiliations:** 1 Division of Cancer Etiology, Department of Population Sciences, Beckman Research Institute, City of Hope, Duarte, California, United States of America; 2 Cancer Prevention Institute of California, Fremont, California, United States of America; 3 Department of Epidemiology, University of Texas, M. D. Anderson Cancer Center, Houston, Texas, United States of America; 4 Department of Environmental and Occupational Health Sciences, School of Public Health, University of Texas Health Science Center, Houston, Texas, United States of America; 5 Department of Epidemiology, School of Public Health, University of Texas Health Science Center, Houston, Texas, United States of America; 6 Department of Nutritional Sciences, Dell Pediatric Research Institute, College of Natural Sciences, Austin, Texas, United States of America; Univesity of Texas Southwestern Medical Center at Dallas, United States of America

## Abstract

Previous results from research on menopausal hormone therapy (MHT) and lung cancer survival have been mixed and most have not studied women who used estrogen therapy (ET) exclusively. We examined the associations between MHT use reported at baseline and lung cancer-specific mortality in the prospective California Teachers Study cohort. Among 727 postmenopausal women diagnosed with lung cancer from 1995 through 2007, 441 women died before January 1, 2008. Hazard Ratios (HR) and 95% Confidence Intervals (CI) for lung-cancer-specific mortality were obtained by fitting multivariable Cox proportional hazards regression models using age in days as the timescale. Among women who used ET exclusively, decreases in lung cancer mortality were observed (HR, 0.69; 95% CI, 0.52–0.93). No association was observed for estrogen plus progestin therapy use. Among former users, shorter duration (<5 years) of exclusive ET use was associated with a decreased risk of lung cancer mortality (HR, 0.56; 95% CI, 0.35–0.89), whereas among recent users, longer duration (>15 years) was associated with a decreased risk (HR, 0.60; 95% CI, 0.38–0.95). Smoking status modified the associations with deceases in lung cancer mortality observed only among current smokers. Exclusive ET use was associated with decreased lung cancer mortality.

## Introduction

Although the rate of lung cancer mortality has been decreasing among men in the United States, it has been stable among women [1]. Among never smokers, women are more likely than men to develop lung cancer [2], [3] and have increased risks of lung cancer at lower levels of cigarette exposure than men [4], [5]. Nevertheless, women diagnosed with lung cancer have better survival and clinical outcomes than men, even within the same strata of stage and histology [6], [7]. The expression of estrogen receptors in both normal and malignant lung tissue, and suggested sex-specific differences in the expression patterning, may be considered as evidence that hormonal mechanisms may drive lung cancer etiology and progression in women [8], [9].

Previous reports of menopausal hormone therapy (MHT) and lung cancer risk have rendered inconsistent results and few studies have investigated the association between MHT and lung cancer mortality. Three studies investigated ever MHT use [3],[10],[11] and four stratified by type of MHT [12]–[15]. Of these, two reported statistically significant decreases in lung cancer survival associated with ever MHT use [10],[12]. Most recently, the Women's Health Initiative (WHI) showed that a combined estrogen plus progestin (E+P) regimen significantly decreased lung cancer survival (hazards ratio (HR), 1.71, 95% confidence interval (CI), 1.16–2.52) [15]. No association was observed for use of estrogen alone (ET); however, these women were not necessarily exclusive users of ET and may have been users of E+P prior to study enrollment. Three studies, in which analyses were stratified by smoking status, suggested that a positive association between MHT use and lung cancer mortality was restricted to ever-smoking women [3],[10],[12].

Gaining a better understanding of the underlying mechanisms of lung cancer progression and survival among women can improve approaches for treatment and provide insights that may improve long-term prognosis. The California Teachers Study (CTS), a largely non-smoking cohort of women, has provided a unique opportunity to study, in detail, the associations between MHT use, specifically exclusive ET use, and lung cancer-specific survival among lung cancer patients diagnosed since the study's inception.

## Materials and Methods

### Ethics Statement

The CTS has been approved by the Institutional Review Boards of the State of California, the Northern California Cancer Center, the Public Health Institute, the University of California, Irvine, the University of Southern California, and the City of Hope National Medical Center [16]. After a complete description of the study to the subjects, written, informed consent was obtained.

### Study Population and Data Collection

The CTS cohort was recruited in 1995–1996 and consists of 133,479 then active and retired female teachers and administrators identified via the California State Teachers Retirement System [17]. The CTS cohort is linked annually with the California Cancer Registry (CCR) to identify incident cancers. Changes of address are obtained through annual mailings, responses from participants, and record linkages with the US Postal Service National Change of Address database. State and national mortality files are used to ascertain date and cause of death for cohort members.

Each CTS participant returned a 16-page, mailed, optically scannable questionnaire at baseline that covered a wide variety of demographics and risk factors related to cancer and women's health, including current MHT use at baseline (recent use) and past MHT use, menopausal status, and cigarette smoking habits. Data were collected separately for estrogens (ET) and progestins (PT), and included ages of first and last use, and duration of use as well as mode of E administration (pill, patch, injection, or vaginal cream).

Menopausal status (premenopausal, perimenopausal, postmenopausal, or unknown menopausal status) was derived at baseline from responses to questions about menstrual periods, duration and timing of estrogen and progestin therapy, age of respondent, and ages at ovarian and uterine surgeries, if relevant. Participants were asked if they had ever smoked 100 or more cigarettes during their lifetimes and, if so, at what ages did they start and stop smoking. Based on their responses, respondents were categorized as never or ever (former and current) smokers. A five-category smoking variable was created based on smoking status and median pack-years; 1) never smokers, 2) former light smokers (pack-years <31.5, the median pack-years for all former smokers), 3) former heavy smokers (pack-years ≥31.5 pack-years), 4) current light smokers (pack-years <45.6, the median pack-years for all current smokers), and 5) current heavy smokers (pack-years ≥45.6 pack-years). Participants were also asked if their parents smoked in the house in which they lived as children and, as an adult, if persons in their households smoked. Based on these responses, respondents were categorized as having no passive smoke exposure, childhood passive smoke exposure only, adult passive smoke exposure only or both childhood and adult passive smoke exposure.

Eligibility for the current analysis required residence in California at baseline to assure coverage by the CCR, and diagnosis of lung cancer during follow up. Only women with no prior history of cancer and whose first cancer diagnosis was lung were included (n = 906). Further, women were excluded, in sequence, if they were premenopausal (n = 63), had no information on smoking (n = 42), or did not provide complete information on MHT (n = 74). A total of 727 eligible postmenopausal women (including 184 never smokers) were diagnosed with lung cancer between 1995 and 2007 and were followed for mortality; 441 of these women died of lung cancer during follow-up through December 31, 2007.

Pathological confirmation of lung cancer diagnoses and tumor stage at diagnosis (localized, regional extension only, regional lymph nodes only, regional extension and lymph nodes or distant metastases) were obtained from CCR records. Lung cancer histology was categorized as small cell lung cancer (SCLC, International Classification of Diseases for Oncology 3, ICD-O-3 morphology codes 8041–8045) and non-small cell lung cancer (NSCLC).

#### Statistical Analyses

Descriptive analyses were conducted to characterize the study population. HRs and 95% CIs for lung-cancer-specific mortality associated with MHT use were obtained by fitting Cox proportional hazards regression models using age in days as the timescale (e.g., where subjects entered the risk set at diagnosis and exited at death/censoring based on their ages at those time points) with adjustment for confounders observed to be significant during model selection and/or biologically relevant including race/ethnicity, smoking status, and tumor stage at diagnosis. Women were followed from the date of lung cancer diagnosis to the first of the following dates: death, moved out of the United States, or December 31, 2007. Women who moved out of the United States (n = 1), who died from a cause other than lung cancer (n = 85), or were alive on December 31, 2007 (n = 200) were censored (at the appropriate time). Kaplan-Meier survival curves and log-rank tests were computed to examine the differences in lung-cancer-specific mortality by MHT use. Stratified analyses were conducted to assess potential effect modifiers, including smoking status, type of hormone therapy used, stage at diagnosis and histology. All statistical analyses were performed using SAS software, version 9.2 (SAS Institute Inc., Cary, NC, USA) and statistical tests were two sided with a Type I error rate of 5%.

## Results

Of the 727 postmenopausal women with lung cancer, 70.3% reported having ever used MHT (n = 507); of those, 20.2% were former MHT users (n = 147) and 50.1% were recent MHT users (n = 364; Table 1). Recent MHT users were slightly younger at diagnosis than never or former users. Most of the women were White (89.3%); however, MHT use did not differ significantly by race/ethnicity. Three quarters of the women were ever smokers (n = 543). Among the ever smoking women, women with no MHT use reported a mean of 39.8 cigarette pack-years, compared with 42.5 pack-years among former MHT users and 38.5 pack-years among recent MHT users. The majority of the women were diagnosed with NSCLC (91.6%) and half had distant metastases at diagnosis (54.1%).

**Table 1:** Age-adjusted baseline characteristics among 727 postmenopausal women in the California Teachers Study diagnosed with lung cancer stratified by history of menopausal hormone therapy (MHT) use.

Characteristic	N (total)	Baseline status of hormone therapy use (estrogen or estrogen plus progestin)		
		Never MHT user	Former MHT user	Current MHT user
No. lung cancer cases	727	216	147	364
Mean age at diagnosis ± SD		75.2±8.1	75.2±9.1	70.8±8.4
Race/ethnicity (%)^1^				
White	649	29.3	20.5	50.2
Other^2^	72	33.3	18.1	48.6
Smoking status (%)				
Never smoker	184	33.1	24.9	42.1
Former smoker	333	29.2	22.0	48.8
Current smoker	210	34.9	18.0	47.2
Mean pack-years ± SD^3^		39.8±26.9	42.5±26.8	38.5±24.4
Cancer Histology (%)				
Non-Small Cell Lung Cancer	666	31.4	22.1	46.5
Small Cell Lung Cancer	61	34.9	16.6	48.5
Stage at diagnosis (%)				
Localized	153	26.8	21.1	52.2
Regional extension only	51	23.9	30.9	45.2
Regional lymph nodes only	73	33.3	20.0	46.8
Regional extension and lymph nodes	33	30.6	21.1	48.3
Distant	365	34.0	21.0	44.9
Unknown	52	36.9	20.7	42.4

1 Race/ethnicity analysis was not adjusted for by age.

2 Other category for race/ethnicity includes: African American, Hispanic, Native American, Asian/Pacific Islander, Mixed and Unknown.

3 Mean pack-years calculated only among ever smoking women.

**Figure 1:**
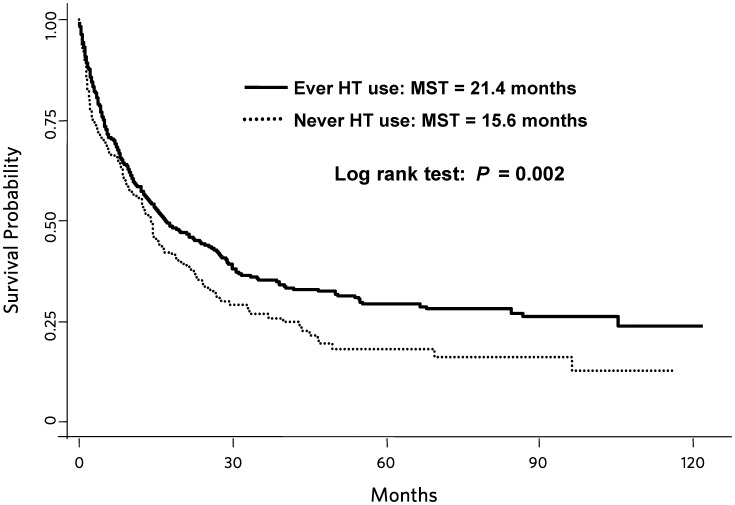
Kaplan-Meier curves of lung cancer-specific survival by ever and never MHT use. Ever MHT use: n = 297 deaths/511 cases; Never MHT use: 144 deaths/216 cases. MST = median survival time.

After adjusting for age, race, smoking and tumor stage, ever MHT use (vs. no use) was associated with a 23% decrease in lung cancer mortality (HR, 0.77; 95% CI, 0.60–0.99; Table 2). The median survival time (MST) for ever users of MHT was 21.4 months versus 15.6 months for never users (log-rank *P* = 0.002; Figure 1). Whether MHT use was former or recent (current at baseline) did not modify the association (HR, 0.68, 95% CI, 0.49–0.95 and HR, 0.82, 95% CI, 0.63–1.08; respectively). Furthermore, longer duration of MHT (ET or E+P) was associated with decreased mortality (<5 years: HR, 0.78, 95% CI, 0.57–1.08; 5–15 years: HR, 0.82, 95% CI, 0.59–1.14; >15 years: HR, 0.68, 95% CI, 0.48–0.94; *P for trend* = 0.034). Former users of MHT who reported their last use of MHT to be ≤5 years prior to baseline were at a 53% decreased risk of lung cancer associated death compared to never users (95% CI, 0.24–0.89). This association was not observed among those who discontinued MHT >5 years before enrolling in the CTS.

**Table 2:** Adjusted^1^ hazard ratios (HR) and 95% confidence intervals (CI) for the association between menopausal hormone therapy (MHT) use and mortality among 727 postmenopausal women diagnosed with lung cancer following enrollment in the California Teachers Study.

MHT Use	N Total	Deaths	Adjusted HR	95% CI
Ever MHT Use				
Never MHT user	216	144	1.00	Ref
Ever MHT user (Former and Recent MHT users)	511	297	0.78	0.61–1.01
Former or Recent MHT Use				
Never MHT user	216	144	1.00	Ref
Former estrogen or estrogen+progestin user	147	90	0.70	0.50–0.97
Recent estrogen or estrogen+progestin user	364	207	0.81	0.62–1.07
Type of MHT Used				
Never MHT user	216	144	1.00	Ref
Former estrogen or estrogen+progestin user	147	90	0.70	0.50–0.97
Recent estrogen therapy	188	109	0.82	0.61–1.12
Recent estrogen+progestin combined therapy	176	98	0.84	0.60–1.17
Duration of MHT Use				
Never MHT user	216	144	1.00	Ref
Ever MHT user, <5 years duration	167	95	0.78	0.57–1.08
Ever MHT user, 5–15 years duration	151	89	0.83	0.60–1.16
Ever MHT user, >15 years duration	151	87	0.69	0.49–0.96
	*P-trend*			*0.049*
Duration of MHT Use				
Never MHT user	216	144	1.00	Ref
Former MHT user				
<5 years duration	97	58	0.75	0.52–1.08
5–15 years duration	31	20	0.65	0.39–1.11
>15 years duration	13	9	1.26	0.54–2.93
	*P-trend*			*0.213*
Recent MHT user				
<5 years duration	70	37	1.00	0.61–1.60
5–15 years duration	120	64	0.73	0.50–1.06
>15 years duration	138	78	0.67	0.47–0.96
	*P-trend*			*0.016*
Years Since Last MHT Use for Former Users				
Never MHT user	216	144	1.00	Ref
Former MHT user, ≤5 years since last use	47	22	0.48	0.25–0.92
Former MHT user, >5 years since last use	100	68	0.81	0.54–1.21

1 Cox proportional hazards regression models using age (in days) as the time metric and stratified by age (in years) with the adjustment for race, a variable combining smoking status and pack-years (never smoker, former light smoker, former heavy smoker, current light smoker, current heavy smoker) and stage.

**All variables measured at baseline.

Decreases in lung cancer mortality were seen for ET use compared to never users among the subset of patients who exclusively used ET (HR, 0.69; 95% CI, 0.52–0.93) (Table 3). The MST for exclusive users of ET was 20.2 months versus 15.6 months for never users (*log-rank P* = 0.008). Among former users, shorter duration (<5 years) of exclusive ET use was associated with a decreased risk of lung cancer mortality (HR, 0.56; 95% CI, 0.35–0.89), whereas among recent users, longer duration (>15 years) was associated with a decreased risk (HR, 0.60; 95% CI, 0.38–0.95). Among former users, a 63% (95% CI, 0.16–0.87) decrease in risk of lung cancer mortality was observed when ET was used exclusively within 5 years of completing the baseline questionnaire; exclusive ET use that ended more than 5 years before baseline was not associated with lung cancer mortality. No statistically significant associations were observed among women who exclusively used E+P, although, with the exception of very recent MHT use, point estimates for this much smaller group of patients were in a similar direction to those for exclusive ET users.

**Table 3:** Adjusted^1^ hazard ratios (HR) and 95% confidence intervals (CI) for the association between menopausal hormone therapy (MHT) use and mortality among 727 postmenopausal women with diagnosed with lung cancer after enrollment in the California Teachers Study stratified by MHT formulation.

	Exclusive ET use		Exclusive E+P use	
MHT Use	N Total//Deaths	HR (95% CI)	N Total//Deaths	HR (95% CI)
Ever MHT Use				
Never MHT user	216/144	Ref	216/144	Ref
Ever MHT user (Former and Recent MHT users)	254/155	0.69 (0.52–0.93)	163/85	0.80 (0.53–1.20)
Former or Recent MHT Use				
Never MHT user	216/144	Ref	216/144	Ref
Former user	101/66	0.69 (0.47–1.01)	35/17	0.89 (0.46–1.72)
Recent user	153/89	0.70 (0.49–0.98)	128/68	0.77 (0.50–1.20)
Duration of MHT Use				
Never MHT user	216/144	Ref	216/144	Ref
Ever MHT user, <5 years duration	87/54	0.59 (0.39–0.87)	74/38	0.92 (0.52–1.62)
Ever MHT user, 6–15 years duration	58/37	0.84 (0.52–1.35)	72/38	0.70 (0.42–1.18)
Ever MHT user, >15 years duration	95/55	0.63 (0.41–0.96)	15/8	0.93 (0.34–2.50)
	*P-trend*	*0.036*		*0.279*
Duration of MHT Use				
Never MHT user	216/144	Ref	216/144	Ref
Former MHT user				
<5 years duration	69/46	0.57 (0.36–0.90)	25/11	0.80 (0.30–2.13)
6–15 years duration	23/15	0.87 (0.42–1.81)	8/5	0.83 (0.20–3.43)
>15 years duration	7/4	0.96 (0.19–4.85)	2/1	0.43 (0.04–4.40)
	*P-trend*	*0.136*		*0.422*
Recent MHT user				
<5 years duration	18/8	0.46 (0.18–1.15)	49/27	0.87 (0.45–1.69)
6–15 years duration	35/22	0.81 (0.43–1.50)	64/33	0.69 (0.40–1.21)
>15 years duration	88/51	0.62 (0.39–0.98)	13/7	1.12 (0.37–3.38)
	*P-trend*	*0.043*		*0.369*
Years Since Last MHT Use for Former Users				
Never MHT user	216/144	Ref	216/144	Ref
Former MHT user, ≤5 years since last use	16/9	0.24 (0.09–0.65)	27/11	0.58 (0.22–1.54)
Former MHT user, >5 years since last use	85/57	0.75 (0.49–1.15)	8/6	1.22 (0.36–4.18)

1 Cox proportional hazards regression models using age (in days) as the time metric and stratified by age (in years) with the adjustment for race, a variable combining smoking status and pack-years (never smoker, former light smoker, former heavy smoker, current light smoker, current heavy smoker) and stage.

**All variables measured at baseline.

Smoking modified the association between MHT use and lung cancer mortality among women diagnosed with lung cancer; specifically, decreases in lung cancer mortality were observed among current smokers (reported currently smoking at baseline), but not among never or former smokers (Table 4). Among baseline current smokers, any MHT use was associated with a 66% decrease in the risk of lung cancer-related death (95% CI, 0.26–0.75) when compared with never MHT use. Similar associations were observed for former versus recent use and E-only versus E+P use. Longer duration of MHT use was associated with decreased lung cancer mortality (<5 years: HR, 0.50, 95% CI, 0.26–0.95; 5–15 years: HR, 0.43, 95% CI, 0.21–0.88; >15 years: HR, 0.39, 95% CI, 0.19–0.81; *P for trend* = 0.034). The number of deaths among women who were exclusive users of E-only or exclusive users of E+P was inadequate too small for meaningful analyses stratified by smoking status.

**Table 4:** Adjusted^1^ hazard ratios (HR) and 95% confidence intervals (CI) for the association between any menopausal hormone therapy (MHT) use and mortality among 727 postmenopausal women diagnosed with lung cancer after enrollment in the California Teachers Study stratified by smoking status at enrollment.

	Never Smokers		Former Smokers		Current Smokers	
MHT Use	N Total/Deaths	HR (95% CI)	N Total/Deaths	HR (95% CI)	N Total/Deaths	HR (95% CI)
Ever MHT Use						
Never MHT user	55/28	Ref	94/62	Ref	67/54	Ref
Ever MHT user (Former and Recent MHT users)	129/62	1.23 (0.58–2.63)	239/138	0.74 (0.50–1.10)	143/97	0.44 (0.26–0.75)
Former or Recent MHT Use						
Never MHT user	55/28	Ref	94/62	Ref	67/54	Ref
Former estrogen or estrogen+progestin user	43/20	0.86 (0.33–2.26)	68/46	0.68 (0.41–1.14)	36/24	0.43 (0.21–0.90)
Recent estrogen or estrogen+progestin user	86/42	1.59 (0.65–3.91)	171/92	0.77 (0.51–1.17)	107/73	0.45 (0.25–0.80)
Type of MHT Used						
Never MHT user	55/28	Ref	94/62	Ref	67/54	Ref
Former estrogen or estrogen+progestin user	43/20	0.86 (0.33–2.27)	68/46	0.68 (0.41–1.14)	36/24	0.43 (0.20–0.89)
Recent estrogen therapy	43/23	1.81 (0.67–4.88)	92/50	0.81 (0.51–1.30)	51/35	0.39 (0.20–0.75)
Recent estrogen+progestin combined therapy	43/19	1.41 (0.47–4.27)	78/42	0.71 (0.41–1.22)	55/37	0.55 (0.27–1.13)
Duration of MHT Use						
Never MHT user	55/28	Ref	94/62	Ref	67/54	Ref
Ever MHT user, <5 years duration	48/20	0.89 (0.34–2.32)	70/45	0.64 (0.38–1.10)	49/30	0.50 (0.26–0.95)
Ever MHT user, 5–15 years duration	42/22	1.54 (0.54–4.38)	70/40	0.70 (0.42–1.19)	39/27	0.43 (0.21–0.88)
Ever MHT user, >15 years duration	30/14	1.72 (0.55–5.35)	77/42	0.73 (0.44–1.21)	44/31	0.39 (0.19–0.81)
	*P-trend*	*0.364*		*0.242*		*0.011*

1 Cox proportional hazards regression models using age (in days) as the time metric and stratified by age (in years) with the adjustment for race and stage.

**All variables measured at baseline.

## Discussion

Associations between MHT and lung cancer previously reported in the literature have been inconsistent and few studies have been able to report results for exclusive ET and exclusive E+P separately. The current study demonstrated that ET use was associated with a statistically significant decrease in lung-cancer-specific mortality. Among former exclusive ET users, decreases in lung cancer mortality were associated with shorter duration (<5 years), whereas among recent exclusive ET users, decreases in lung cancer mortality were associated with longer duration (>15 years). Furthermore, among former users, a decrease in lung cancer mortality was observed for exclusive ET use within 5 years prior to baseline, but not for ET use that ended earlier.

Two other studies published to date examined the association between ET use and lung cancer mortality. Ettinger et al. [13] investigated long-term postmenopausal ET use and mortality (overall and lung cancer specific) comparing 232 ET users who began use within 3 years of menopause and used ET for at least 5 years to 222 age-matched non-users. Long-term ET use was not associated with lung cancer mortality (HR, 0.22, 95% CI, 0.04–1.15) [13]; however, the study did not investigate short-term use or timing of use (recent or former), possibly because few deaths occurred in this small study. More recently, Chlebowski et al. [15] reported on the estrogen-alone results of the WHI clinical trial where 10,739 postmenopausal women were randomized and followed for a mean of 7.9 years. The use of conjugated equine estrogen (CEE) alone did not decrease lung cancer mortality; however, the ET users in the WHI trial [15] were not necessarily exclusive users of ET prior to entry into the study and may have used E+P previously.

Research has shown that after a traumatic injury, elderly populations generally have a poorer prognosis than younger populations [18]. It has been hypothesized that this age-dependent trend is influenced by a hyper-inflammatory state coined as ‘inflamm-aging,’ which is characterized by the overproduction of proinflammatory cytokines and, consequently, immunosuppression [18]. Recent evidence has suggested that at physiological levels, estrogen may help to boost the immune system and attenuate aberrant production of pro-inflammatory cytokines [18]; and the results of the current study may reflect this phenomenon. Inflammation has been identified as a major player in lung carcinogenesis with recent research reporting key inflammation genes associated with lung cancer survival among patients who have radiation-induced tissue damage [19]. If estrogen serves as an anti-inflammatory agent, it may be that lung cancer patients in a chronic inflammatory state, who use MHT in the form of ET alone are attenuating the inflammatory responses, thus, boosting their immune systems and, subsequently, increasing their survival [19]. It would follow, then, that this association would only be observed among recent users of ET or former users of ET who stopped use within 5 years of baseline, since timing of estrogen usage was closest to the chronic inflammatory state; whereas the anti-inflammatory effect of estrogen may have lost its effectiveness among former users.

The current study did not observe a statistically significant association between exclusive E+P use and lung cancer mortality, although, with the exception of very recent MHT use, point estimates for this much smaller group of patients were in a similar direction to those for exclusive ET users. Similarly, the Heart and Estrogen/Progestin Replacement Study (HERS) did not observe an association [14]. However, the WHI clinical trial of CEE plus MPA observed that women using CEE plus MPA who developed lung cancer after randomization had a higher risk of lung cancer mortality (HR, 1.71, 95% CI, 1.16–2.52) than women diagnosed with lung cancer in the placebo group [12].

Smoking modified the association between MHT use and lung cancer mortality. Decreases in lung cancer mortality were observed among current, but not among never or former smokers. Among women diagnosed with lung cancer who had a history of smoking, Ganti et al. [10] observed an MST of 73 months for never users of MHT compared to 39 months for ever users of MHT (Log-rank p-value = 0.03). Among never smokers, the MST did not differ by MHT status [10]. Conversely, Huang et al. [3] observed the opposite association among ever smokers diagnosed with lung cancer; i.e., ever users of MHT had an MST of 16.2 months compared to 10.4 months among never users (Log-rank p-value = 0.04) [3]. Chlebowski et al. [12] further stratified smoking status by former and current use. Among former smokers, E+P (CEE plus MPA group) was associated with an 89% increase in lung cancer mortality (95% CI, 1.04–3.45) [12].The association was not observed among current-smoking women.

Several biological mechanisms may explain the observed differences by smoking status. Similar to above, it may be that estrogen is counteracting the proinflammatory effects of cigarette smoking that would have otherwise decreased survival for women who were current smokers at baseline. Exposure to cigarette smoke has been well established as a tumor initiator [19] and, more recently, it has been observed as a tumor promoter following malignant transformation [19]. Furthermore, it has been shown that the tumor promotion is driven by inflammation acting through signaling pathways increasing production of proinflammatory substances [20]. Takahashi et al. [19] noted that if inflammation markers can be identified in human lung cancer, the use of anti-inflammatory drugs may improve prognosis for lung cancer patients, specifically those diagnosed at earlier stages. As indicated previously, estrogen may be one of these agents. Lastly, estrogen receptors are expressed in both normal and malignant lung tissue [8],[9],[21] and it is known that estrogen has the ability to bind to substrates other than those specified for estrogen [22],[23]. It has been hypothesized that carcinogens in cigarette smoke may preferentially bind to estrogen receptors, thus inhibiting their activation and carcinogenic potential [11]. Additionally, recent studies have identified estrogen metabolism-related gene expression changes present in the lung tissue of mice when exposed to tobacco smoke [24]. Future studies should investigate these pathways in humans, specifically ever-smoking wome, since they could directly impact the effect of MHT. One limitation of the current study is that despite careful consideration of smoking status, residual confounding from smoking may exist and the lower risk of lung cancer mortality may reflect unmeasured differences in smoking habits such as differences in smoking intensity. The current study used a five category smoking variable based on smoking status and median pack-years within each smoking group (never; former: light, heavy; current, light, heavy) in multivariable analyses to control not only for smoking status, but also for smoking intensity. Another limitation is that the current study collected MHT use information up to enrollment in the cohort (ranging from less than 1 year to 10 years before lung cancer diagnosis) and did not incorporate MHT use after recruitment into our exposure variables. Third, the small number of deaths in several subgroups limited the ability to examine associations in finely stratified analyses. For example, lung cancer cases who were recent MHT users were diagnosed at a lower stage than those who were never users of MHT, which may in part explain the observed differences in survival; the 5-year survivorship for patients diagnosed with stage 1 disease is 58–73% versus 9–24% for patients diagnosed with stage 3 disease. It is possible that women receiving MHT were more likely to be under active medical management and thus more likely to have any symptoms of lung cancer receive medical work up and diagnosis. A larger study with adequate power to study these groups independently is warranted. A further limitation is the lack of detailed treatment data; however, since lung cancer treatment is based substantially on tumor stage, tumor stage at diagnosis was a proxy for treatment in our statistical models and adjusted for accordingly.

The results from the current study suggest exclusive ET use decreases lung cancer mortality. Given the substantial clinical significance that estrogen may have as an anti-inflammatory therapy or intervention in lung cancer patients, specifically among those with early stage disease, validation of these findings and detailed investigation of the causal mechanisms driving the associations are needed, including studies that explore the potential anti-inflammatory effects of estrogen and the interaction between smoking and these exposures.

## References

[1] Jemal A, Siegel R, Ward E, Hao Y, Xu J, et al (2009) Cancer statistics, 2009. CA Cancer J Clin 59: 225–249. 19474385 10.3322/caac.20006

[2] Ayeni O, Robinson A (2009) Hormone replacement therapy and outcomes for women with non-small-cell lung cancer: can an association be confirmed? Curr Oncol 16: 21–25. 10.3747/co.v16i3.302 PMC2695714 19526081

[3] Huang B, Carloss H, Wyatt SW, Riley E (2009) Hormone replacement therapy and survival in lung cancer in postmenopausal women in a rural population. Cancer 115: 4167–4175. 19526591 10.1002/cncr.24475

[4] Wakelee HA, Chang ET, Gomez SL, Keegan TH, Feskanich D, et al (2007) Lung cancer incidence in never smokers. J Clin Oncol 25: 472–478. 17290054 10.1200/JCO.2006.07.2983 PMC2764546

[5] Sekine I, Sumi M, Ito Y, Tanai C, Nokihara H, et al (2009) Gender difference in treatment outcomes in patients with stage III non-small cell lung cancer receiving concurrent chemoradiotherapy. Jpn J Clin Oncol 39: 707–712. 19692418 10.1093/jjco/hyp095

[6] Thomas L, Doyle LA, Edelman MJ (2005) Lung cancer in women: emerging differences in epidemiology, biology, and therapy. Chest 128: 370–381. 16002959 10.1378/chest.128.1.370

[7] Henschke CI, Yip R, Miettinen OS (2006) Women's susceptibility to tobacco carcinogens and survival after diagnosis of lung cancer. Jama 296: 180–184. 16835423 10.1001/jama.296.2.180

[8] Siegfried JM (2001) Women and lung cancer: does oestrogen play a role? Lancet Oncol 2: 506–513. 11905727 10.1016/S1470-2045(01)00457-0

[9] Subramanian J, Govindan R (2007) Lung cancer in never smokers: a review. J Clin Oncol 25: 561–570. 17290066 10.1200/JCO.2006.06.8015

[10] Ganti AK, Sahmoun AE, Panwalkar AW, Tendulkar KK, Potti A (2006) Hormone replacement therapy is associated with decreased survival in women with lung cancer. J Clin Oncol 24: 59–63. 16314616 10.1200/JCO.2005.02.9827

[11] Schabath MB, Wu X, Vassilopoulou-Sellin R, Vaporciyan AA, Spitz MR (2004) Hormone replacement therapy and lung cancer risk: a case-control analysis. Clin Cancer Res 10: 113–123. 14734459 10.1158/1078-0432.ccr-0911-3

[12] Chlebowski RT, Schwartz AG, Wakelee H, Anderson GL, Stefanick ML, et al (2009) Oestrogen plus progestin and lung cancer in postmenopausal women (Women's Health Initiative trial): a post-hoc analysis of a randomised controlled trial. Lancet 374: 1243–1251. 19767090 10.1016/S0140-6736(09)61526-9 PMC2995490

[13] Ettinger B, Friedman GD, Bush T, Quesenberry CP Jr (1996) Reduced mortality associated with long-term postmenopausal estrogen therapy. Obstet Gynecol 87: 6–12. 8532268 10.1016/0029-7844(95)00358-4

[14] Hulley S, Furberg C, Barrett-Connor E, Cauley J, Grady D, et al (2002) Noncardiovascular disease outcomes during 6.8 years of hormone therapy: Heart and Estrogen/progestin Replacement Study follow-up (HERS II). Jama 288: 58–66. 12090863 10.1001/jama.288.1.58

[15] Chlebowski RT, Anderson GL, Manson JE, Schwartz AG, Wakelee H, et al (2010) Lung cancer among postmenopausal women treated with estrogen alone in the women's health initiative randomized trial. J Natl Cancer Inst 102: 1413–1421. 20709992 10.1093/jnci/djq285 PMC2943522

[16] Horn-Ross PL, Canchola AJ, West DW, Stewart SL, Bernstein L, et al (2004) Patterns of alcohol consumption and breast cancer risk in the California Teachers Study cohort. Cancer Epidemiol Biomarkers Prev 13: 405–411. 15006916

[17] Bernstein L, Allen M, Anton-Culver H, Deapen D, Horn-Ross PL, et al (2002) High breast cancer incidence rates among California teachers: results from the California Teachers Study (United States). Cancer Causes Control 13: 625–635. 12296510 10.1023/a:1019552126105

[18] Kovacs EJ (2005) Aging, traumatic injury, and estrogen treatment. Exp Gerontol 40: 549–555. 15975753 10.1016/j.exger.2005.04.009

[19] Takahashi H, Ogata H, Nishigaki R, Broide DH, Karin M (2010) Tobacco Smoke Promotes Lung Tumorigenesis by Triggering IKKbeta- and JNK1-Dependent Inflammation. Cancer Cell 17: 89–97. 20129250 10.1016/j.ccr.2009.12.008 PMC2818776

[20] Karin M, Greten FR (2005) NF-kappaB: linking inflammation and immunity to cancer development and progression. Nat Rev Immunol 5: 749–759. 16175180 10.1038/nri1703

[21] Niikawa H, Suzuki T, Miki Y, Suzuki S, Nagasaki S, et al (2008) Intratumoral estrogens and estrogen receptors in human non-small cell lung carcinoma. Clin Cancer Res 14: 4417–4426. 18579664 10.1158/1078-0432.CCR-07-1950

[22] Flototto T, Djahansouzi S, Glaser M, Hanstein B, Niederacher D, et al (2001) Hormones and hormone antagonists: mechanisms of action in carcinogenesis of endometrial and breast cancer. Horm Metab Res 33: 451–457. 11544557 10.1055/s-2001-16936

[23] Pike AC, Brzozowski AM, Hubbard RE, Bonn T, Thorsell AG, et al (1999) Structure of the ligand-binding domain of oestrogen receptor beta in the presence of a partial agonist and a full antagonist. EMBO J 18: 4608–4618. 10469641 10.1093/emboj/18.17.4608 PMC1171535

[24] Meireles SI, Esteves GH, Hirata R Jr, Peri S, Devarajan K, et al (2010) Early changes in gene expression induced by tobacco smoke: Evidence for the importance of estrogen within lung tissue. Cancer Prev Res (Phila) 3: 707–717. 20515954 10.1158/1940-6207.CAPR-09-0162 PMC2896420

